# Supplementary material for: Cloning and Characterization of Three Sugar Metabolism Genes (LBGAE, LBGALA, and LBMS) Regulated in Response to Elevated CO2 in Goji Berry (Lycium barbarum L.)
Source: Plants (Basel). 2021 Feb 7;10(2):321. doi: 10.3390/plants10020321 (PMC7914792; doi:10.3390/plants10020321)
Supplement: Supplementary file 1 [file plants-10-00321-s001.pdf]

Supplemental data

Cloning and characterization of three sugar metabolism genes (*LBGAE*, *LBGALA*, and *LBMS*) regulated in response to elevated CO_2 in goji berry (*Lycium barbarum* L.)

Yaping Ma [1,2,3], Mura Jyostna Devi [3,4,5*], Vangimalla R. Reddy [3], Lihua Song [2,3], Handong Gao [1] and Bing Cao [2,*]

1 College of Forestry, Nanjing Forestry University, Nanjing 210037, China; YapingMa@njfu.edu.cn (Y.M.); gaohd@njfu.edu.cn (H.G.)
2 School of Agriculture, Ningxia University, Yinchuan 750021, China; slh382@126.com (L.S.)
3 USDA-ARS, Adaptive Cropping Systems Laboratory, 10300 Baltimore Ave, Beltsville, MD 20705, USA;Vangimalla.Reddy@ars.usda.gov (V.R.)
4 USDA-ARS, Vegetable Crops Research Unit, Madison, WI 53706, USA
5 Department of Horticulture, University of Wisconsin-Madison, Madison 53705, WI, USA; Jyostna.mura@usda.gov (M.J.D.)

* Correspondence: Jyostna.mura@usda.gov (M.J.D.); bingcao2006@126.com (B.C.)

List of materials:

Figure S1 Complete nucleotide and deduced amino acid sequence of cDNA of gene *LBGAE*, *LBGALA*, *LBMS*

Figure S2 Prediction of protein secondary structure of *LBGAE*, *LBGALA* and *LBMS* sequences.

Figure S3 Multiple sequence alignment of *LBGAE*, *LBGALA* and *LBMS* amino acid sequences with other known species.

Table S1. Primer sequences for ORF sequences cloning and qPCR

(a)

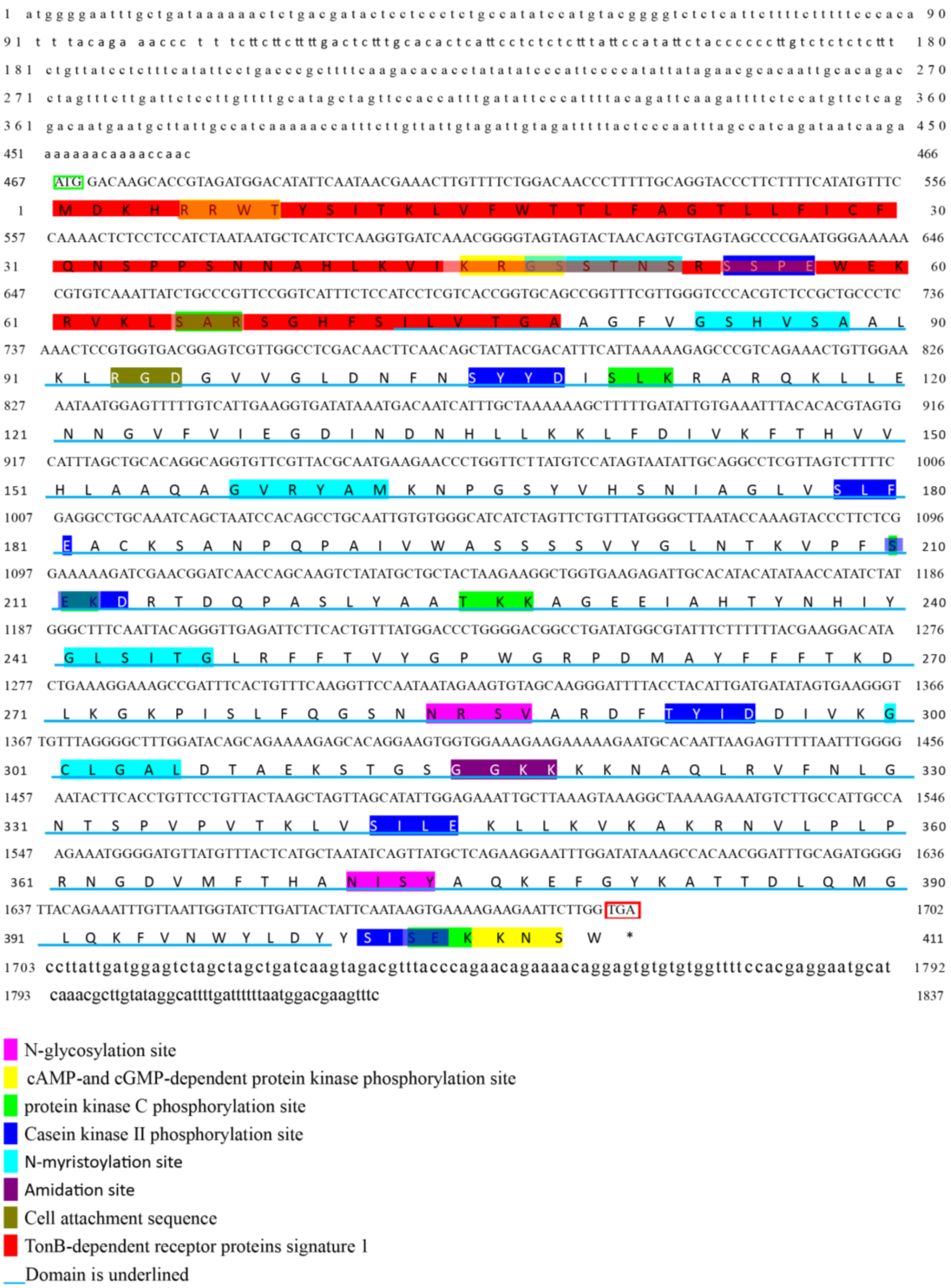
1 atggggaatttgctgataaaaaactctgacgatactcctccctctgccatatccatgtacggggtctctcattcttttctttttcccaca 90
91 t t tacaga aaccc t t tcttcttctttgactctttgcacactcattcctctctcttattccatattctaccccccttgtctctctcttt 180
181 ctgttatcctctttcatattcctgacccgcttttcaagacacacctatatatcccattccccatattatagaacgcacaattgcacagac 270
271 ctagtttcttgattctccttgttttgcatagctagttccaccatttgatattcccattttacagattcaagattttctccatgttctcag 360
361 gacaatgaatgcttattgccatcaaaaaccatttcttgttattgtagattgtagatttttactcccaatttagccatcagataatcaaga 450
451 aaaaaacaaaaccaac 466
467 ATG GACAAGCACCGTAGATGGACATATTCAATAACGAAACTTGTTTTCTGGACAACCCTTTTTGCAGGTACCCTTCTTTTCATATGTTTC 556
1 M D K H R R W T Y S I T K L V F W T T L F A G T L L F I C F 30
557 CAAAACTCTCCTCCATCTAATAATGCTCATCTCAAGGTGATCAAACGGGGTAGTAGTACTAACAGTCGTAGTAGCCCCGAATGGGAAAAA 646
31 Q N S P P S N N A H L K V I K R G S S T N S R S S P E W E K 60
647 CGTGTCAAATTATCTGCCCGTTCCGGTCATTTCTCCATCCTCGTCACCGGTGCAGCCGGTTTCGTTGGGTCCCACGTCTCCGCTGCCCTC 736
61 R V K L S A R S G H F S I L V T G A A G F V G S H V S A A L 90
737 AAACTCCGTGGTGACGGAGTCGTTGGCCTCGACAACTTCAACAGCTATTACGACATTTCATTAAAAAGAGCCCGTCAGAAACTGTTGGAA 826
91 K L R G D G V V G L D N F N S Y Y D I S L K R A R Q K L L E 120
827 AATAATGGAGTTTTTGTCATTGAAGGTGATATAAATGACAATCATTTGCTAAAAAAGCTTTTTGATATTGTGAAATTTACACACGTAGTG 916
121 N N G V F V I E G D I N D N H L L K K L F D I V K F T H V V 150
917 CATTTAGCTGCACAGGCAGGTGTTCGTTACGCAATGAAGAACCCTGGTTCTTATGTCCATAGTAATATTGCAGGCCTCGTTAGTCTTTTC 1006
151 H L A A Q A G V R Y A M K N P G S Y V H S N I A G L V S L F 180
1007 GAGGCCTGCAAATCAGCTAATCCACAGCCTGCAATTGTGTGGGCATCATCTAGTTCTGTTTATGGGCTTAATACCAAAGTACCCTTCTCG 1096
181 E A C K S A N P Q P A I V W A S S S S V Y G L N T K V P F S 210
1097 GAAAAAGATCGAACGGATCAACCAGCAAGTCTATATGCTGCTACTAAGAAGGCTGGTGAAGAGATTGCACATACATATAACCATATCTAT 1186
211 E K D R T D Q P A S L Y A A T K K A G E E I A H T Y N H I Y 240
1187 GGGCTTTCAATTACAGGGTTGAGATTCTTCACTGTTTATGGACCCTGGGGACGGCCTGATATGGCGTATTTCTTTTTTACGAAGGACATA 1276
241 G L S I T G L R F F T V Y G P W G R P D M A Y F F F T K D 270
1277 CTGAAAGGAAAGCCGATTTCACTGTTTCAAGGTTCCAATAATAGAAGTGTAGCAAGGGATTTTACCTACATTGATGATATAGTGAAGGGT 1366
271 L K G K P I S L F Q G S N N R S V A R D F T Y I D D I V K G 300
1367 TGTTTAGGGGCTTTGGATACAGCAGAAAAGAGCACAGGAAGTGGTGGAAAGAAGAAAAAGAATGCACAATTAAGAGTTTTTAATTTGGGG 1456
301 C L G A L D T A E K S T G S G G K K K K N A Q L R V F N L G 330
1457 AATACTTCACCTGTTCCTGTTACTAAGCTAGTTAGCATATTGGAGAAATTGCTTAAAGTAAAGGCTAAAAGAAATGTCTTGCCATTGCCA 1546
331 N T S P V P V T K L V S I L E K L L K V K A K R N V L P L P 360
1547 AGAAATGGGGATGTTATGTTTACTCATGCTAATATCAGTTATGCTCAGAAGGAATTTGGATATAAAGCCACAACGGATTTGCAGATGGGG 1636
361 R N G D V M F T H A N I S Y A Q K E F G Y K A T T D L Q M G 390
1637 TTACAGAAATTTGTTAATTGGTATCTTGATTACTATTCAATAAGTGAAAAGAAGAATTCTTGG TGA 1702
391 L Q K F V N W Y L D Y Y S I S E K K N S W * 411
1703 ccttattgatggagtctagctagctgatcaagtagacgtttacccagaacagaaaacaggagtgtgtgtggttttccacgaggaatgcat 1792
1793 caaacgcttgtataggcattttgattttttaatggacgaagtttc 1837
N-glycosylation site
cAMP-and cGMP-dependent protein kinase phosphorylation site
protein kinase C phosphorylation site
Casein kinase II phosphorylation site
N-myristoylation site
Amidation site
Cell attachment sequence
TonB-dependent receptor proteins signature 1
Domain is underlined

(b)

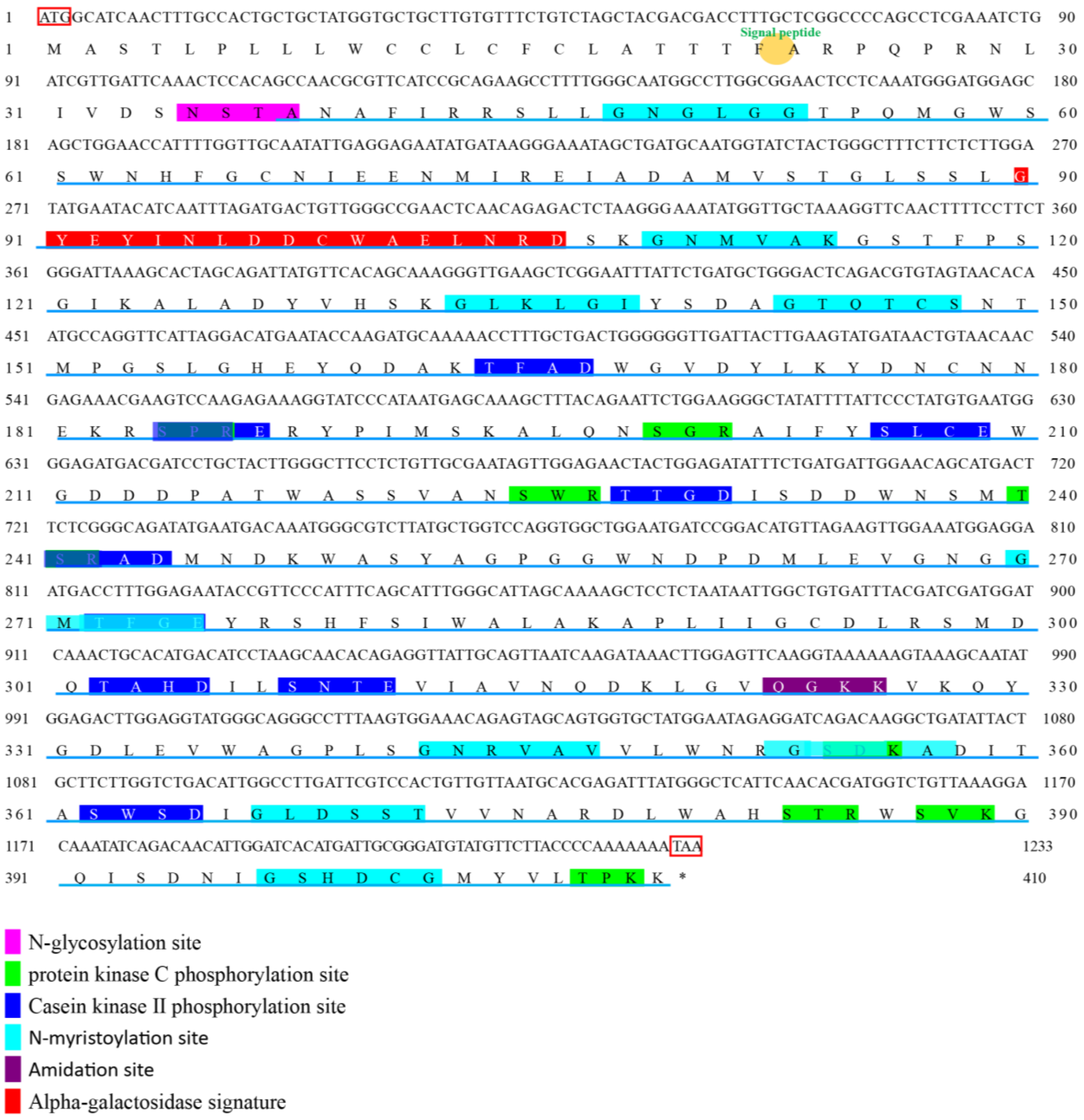
1 ATGGCATCAACTTTGCCACTGCTGCTATGGTGCTGCTTGTGTTTCTGTCTAGCTACGACGACCTTTGCTCGGCCCCAGCCTCGAAATCTG 90
Signal peptide
1 M A S T L P L L L W C C L C F C L A T T T F A R P Q P R N L 30
91 ATCGTTGATTCAAACTCCACAGCCAACGCGTTCATCCGCAGAAGCCTTTTGGGCAATGGCCTTGGCGGAACTCCTCAAATGGGATGGAGC 180
31 I V D S N S T A N A F I R R S L L G N G L G G T P Q M G W S 60
181 AGCTGGAACCATTTTGGTTGCAATATTGAGGAGAATATGATAAGGGAAATAGCTGATGCAATGGTATCTACTGGGCTTTCTTCTCTTGGA 270
61 S W N H F G C N I E E N M I R E I A D A M V S T G L S S L G 90
271 TATGAATACATCAATTTAGATGACTGTTGGGCCGAACTCAACAGAGACTCTAAGGGAAATATGGTTGCTAAAGGTTCAACTTTTCCTTCT 360
91 Y E Y I N L D D C W A E L N R D S K G N M V A K G S T F P S 120
361 GGGATTAAAGCACTAGCAGATTATGTTCACAGCAAAGGGTTGAAGCTCGGAATTTATTCTGATGCTGGGACTCAGACGTGTAGTAACACA 450
121 G I K A L A D Y V H S K G L K L G I Y S D A G T Q T C S N T 150
451 ATGCCAGGTTCATTAGGACATGAATACCAAGATGCAAAAACCTTTGCTGACTGGGGGGTTGATTACTTGAAGTATGATAACTGTAACAAC 540
151 M P G S L G H E Y Q D A K T F A D W G V D Y L K Y D N C N N 180
541 GAGAAACGAAGTCCAAGAGAAAGGTATCCCATAATGAGCAAAGCTTTACAGAATTCTGGAAGGGCTATATTTTATTCCCTATGTGAATGG 630
181 E K R S P R E R Y P I M S K A L Q N S G R A I F Y S L C E W 210
631 GGAGATGACGATCCTGCTACTTGGGCTTCCTCTGTTGCGAATAGTTGGAGAACTACTGGAGATATTTCTGATGATTGGAACAGCATGACT 720
211 G D D D P A T W A S S V A N S W R T T G D I S D D W N S M T 240
721 TCTCGGGCAGATATGAATGACAAATGGGCGTCTTATGCTGGTCCAGGTGGCTGGAATGATCCGGACATGTTAGAAGTTGGAAATGGAGGA 810
241 S R A D M N D K W A S Y A G P G G W N D P D M L E V G N G G 270
811 ATGACCTTTGGAGAATACCGTTCCCATTTCAGCATTTGGGCATTAGCAAAAGCTCCTCTAATAATTGGCTGTGATTTACGATCGATGGAT 900
271 M T F G E Y R S H F S I W A L A K A P L I I G C D L R S M D 300
911 CAAACTGCACATGACATCCTAAGCAACACAGAGGTTATTGCAGTTAATCAAGATAAACTTGGAGTTCAAGGTAAAAAAGTAAAGCAATAT 990
301 Q T A H D I L S N T E V I A V N Q D K L G V Q G K K V K Q Y 330
991 GGAGACTTGGAGGTATGGGCAGGGCCTTTAAGTGGAAACAGAGTAGCAGTGGTGCTATGGAATAGAGGATCAGACAAGGCTGATATTACT 1080
331 G D L E V W A G P L S G N R V A V V L W N R G S D K A D I T 360
1081 GCTTCTTGGTCTGACATTGGCCTTGATTCGTCCACTGTTGTTAATGCACGAGATTTATGGGCTCATTCAACACGATGGTCTGTTAAAGGA 1170
361 A S W S D I G L D S S T V V N A R D L W A H S T R W S V K G 390
1171 CAAATATCAGACAACATTGGATCACATGATTGCGGGATGTATGTTCTTACCCCAAAAAAATAA 1233
391 Q I S D N I G S H D C G M Y V L T P K K * 410
N-glycosylation site
protein kinase C phosphorylation site
Casein kinase II phosphorylation site
N-myristoylation site
Amidation site
Alpha-galactosidase signature

(c)

1 cacacattgcagaaaactccaaagcatgccacttgtcaaagaagaaagtttaataacttcaatatatcctaacaaattgcaactcccgag 90

91 atcgttgattcaaactccacagccaacgcgttcatccgcagaagccttttgggcaatggccttggcggaactcctcaaatgggatggagc 180

181 catctgacgcacataacgccatggtttttctaattatagacccataacgataagccttacctccattaaattttaaaaaaagaatatcat 270

271 aaaatcagtagctatccacatattataaatttcaccaatccaaacttcctgtttcccctctttatattgcagccatctgctagctagatt 360

361 gttgagaaataataagtagtaattaccaaaagaaggaaaa 400

401 ATG GTTAGCTTTGAGACATTCCTTCAACCCAATAACCCTGTTCAAAGAAAGAGTAGTGTAAAGATGGGTTATGATGTGCCTGAAGGAGTG 490

1 M V S F E T F L Q P N N P V Q R K S S V K M G Y D V P E G V 30

491 GACATTAGGGGAAGATATGATCCTGAATTTTCCAAGATTTTGGCAAGGGATGCTTTGCAATTTGTAGCTGATTTGCAGAGG GAATTCAGG 580

31 D I R G R Y D P E F S K I L A R D A L Q F V A D L Q R E F R 60

581 AACCACATTAAGTATGCTATGGAGTGTAGGAAAGAGGCTAAGAAGAGGTATAATAATGGAGGTTTGCCAGGTTTTGATCCGGCAACCAAA 670

61 N H I K Y A M E C R K E A K K R Y N N G G L P G F D P A T K 90

671 TATATTAGGGATGGTGAGTGGGTGTGTGCTCCTGTTCCGCAGGCGGTGGCTGATCGGAGGGTGGAGATTACTGGTCCAGTTGAGAGGAAG 760

91 Y I R D G E W V C A P V P Q A V A D R R V E I T G P V E R K 120

761 ATGGTCATCAATGCCCTTAATTCTGGAGCCAAGGTTTTCATGGCAGACTTTGAAGATGCACTGTCACCAAACTGGGAGAATCTAATGAGA 850

121 M V I N A L N S G A K V F M A D F E D A L S P N W E N L M R 150

851 GGCCATGTAAATTTGAGGGATGCAGTGAATGGAACAATAACATTCCATGATCAAGCCAGAAACAAACTGTATAAACTGAATAATCAGATA 940

151 G H V N L R D A V N G T I T F H D Q A R N K L Y K L N N Q I 180

941 GCTAAGCTATTTGTGCGTCCAAGAGGGTGGCATTTGCCAGAAGCTCACGTCTTCATTGATGGTGAGCCTGCCACTGGTTGCCTTGTTGAC 1030

181 A K L F V R P R G W H L P E A H V F I D G E P A T G C L V D 210

1031 TTCGGTCTCTACTTTTTCCACAACTATGCCAACTTCCGCAAGACCCAAGGACAGGGCTTTGGACCCTTTTTCTATCTTCCCAAAATGGAA 1120

211 F G L Y F F H N Y A N F R K T Q G Q G F G P F F Y L P K M E 240

1121 CACTCTAGGGAAGCTAGAATATGGAACAATGTGTTTGACAGGGCGGAGAAATGGGCTGGAATTGAGAAAGGAAGCATTAGGGCCACTGTC 1210

241 H S R E A R I W N N V F D R A E K W A G I E K G S I R A T V 270

1211 CTAATTGAAACACTTCCAGCAGTGTTTCAAATGAATGAAATTTTGTATGAACTGAGGGACCATTCTGTTGGCCTCAACTGTGGTAGATGG 1300

271 L I E T L P A V F Q M N E I L Y E L R D H S V G L N C G R W 300

1301 GATTACATCTTCAGCTATGTCAAGACTTTCCAAGCTCACCCTGATCGCCTGCTACCCGATAGGGTTCTAGTTGGCATGGGTCAGCACTTT 1390

301 D Y I F S Y V K T F Q A H P D R L L P D R V L V G M G Q H F 330

1391 ATGAGGAGTTACTCTGACTTGCTCATCCACACTTGTCATAAGCGCGGTGTCCACGCTATGGGAGGCATGGCAGCTCAGATTCCTATCAGA 1480

331 M R S Y S D L L I H T C H K R G V H A M G G M A A Q I P I R 360

1481 GATGATGCAGCAGCTAATGAGGCAGCATTGGAACTTGTAAGGAAGGATAAGCTGAGAGAAGTGAAGGCAGGGCATGATGGAACATGGGCA 1570

361 D D A A A N E A A L E L V R K D K L R E V K A G H D G T W A 390

1571 GCTCACCCTGGCCTAATCCCAGCCTGCATGGAAGTCTTCACCAGTAACATGACCAATGCACCTAACCAAATCCATTCCATGAAGCGCCAA 1660

391 A H P G L I P A C M E V F T S N M T N A P N Q I H S M K R Q 420

1661 GATGCATCGGTCCTAGTTGAAGAAGACCTGCTGCAGAGGCCGAGAGGGGTCCGAACCATGGAGGGTCTCCGGCTGAACACCCGAGTGGGA 1750

421 D A S V L V E E D L L Q R P R G V R T M E G L R L N T R V G 450

1751 ATCCAGTACTTAGCAGCCTGGCTGACTGGGGCTGGTTCTGTCCCACTTTACAACCTCATGGAAGATGCCGCAACAGCTGAGATTAGTAGA 1840

451 I Q Y L A A W L T G A G S V P L Y N L M E D A A T A E I S R 480

1841 GTCCAGAACTGGCAGTGGCTGAAATATGGAGTGGAATTGGATGGAGATGGACTTGGGGTGAAGGTGAACTTGGACCTGTTTGGAAGAGTG 1930

481 V Q N W Q W L K Y G V E L D G D G L G V K V N L D L F G R V 510

1931 GTTGAAGAAGAAATGGCTAGGATTGAGAGAGAAGTGGGAAAGGAGAAATTCAAGAAAGGAATGTACAAGGAGGCATGCAAGTTATTCACA 2020

511 V E E E M A R I E R E V G K E K F K K G M Y K E A C K L F T 540

2021 AGGCAATGCACTGCACCAGTCTTGGATGACTTTCTGACTCTTGATGCCTACAATAACATTGTCATGCATCATCCTATTGGATTGTCCCGG 2110

541 R Q C T A P V L D D F L T L D A Y N N I V M H H P I G L S R 570

2111 CTC TAA 2116

571 I *

2117 atttcgcagcttttgctgtaagataaatcgggtgtctctatattgtgactagtgctatcttccctgcaataatgataggaaatggaagtt 2206

2207 cgcctttgaggagatagttgattatccaaaggctctgtttttcagctgtgcttttcaatcaatctgttatttgtatttgtgagtactgc 2296

2297 tatctttccctgtaataataaaggcaattgttcatttattgtcctacttttccaatacatacgaacctctcatttcaacgtgcatatat 2386

2387 cttcttgtacttgttaaattttcgctgaattcagaacactacaggctatggattagtttgaac 2449

Figure S1. Complete nucleotide and deduced amino acid sequence of cDNA of gene *LBGAE* (*Lycium barbarum* UDP-glucuronate 4-epimerase) (**a**), *LBGALA* (*Lycium barbarum* alpha-galactosidase) (**b**), and *LBMS* (*Lycium barbarum* malate synthase) (**c**). The amino acid residues are represented by one-letter symbols. The open reading frame (ORF), from the initiation codon ATG to the termination codon TAA is to be uppercase. The domain is defined by a straight blue line. Motif-scan predictive protein site, different colors represent different protein sites, including casein kinase II phosphorylation sites, N-glycosylation sites, cAMP-and cGMP-dependent protein kinase phosphorylation sites, protein kinase C phosphorylation sites, N-myristoylation sites, Amidation site, cell attachment sequence, andTonB-dependent receptor proteins signature.

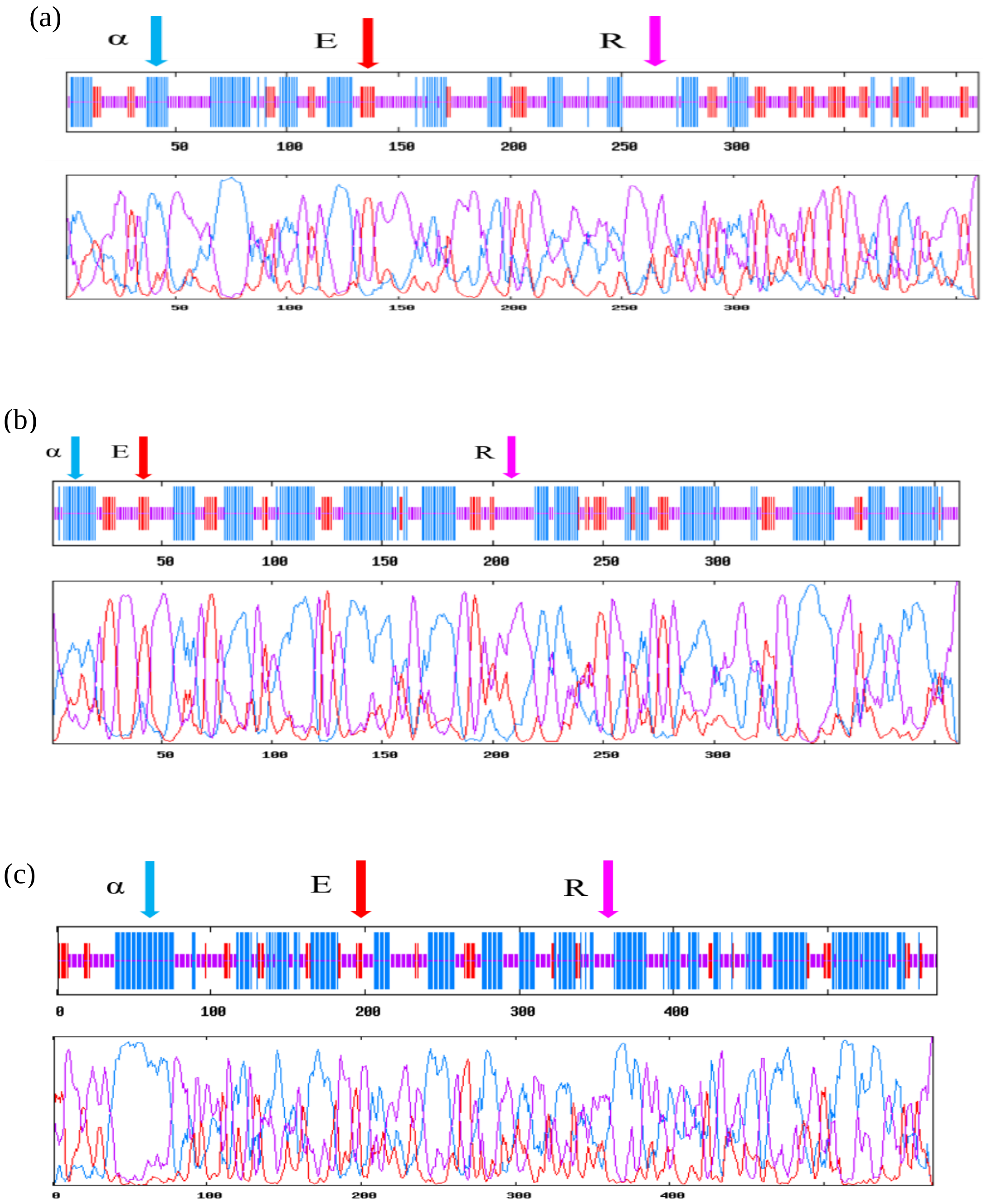

Figure S2 Prediction of protein secondary structure of *LBGAE* (a), *LBGALA*(b) and *LBMS*(c) sequences. The secondary structure of the three genes is mainly composed of Alpha helix (α), Random coil (R) and Extended strand(E).

(a)

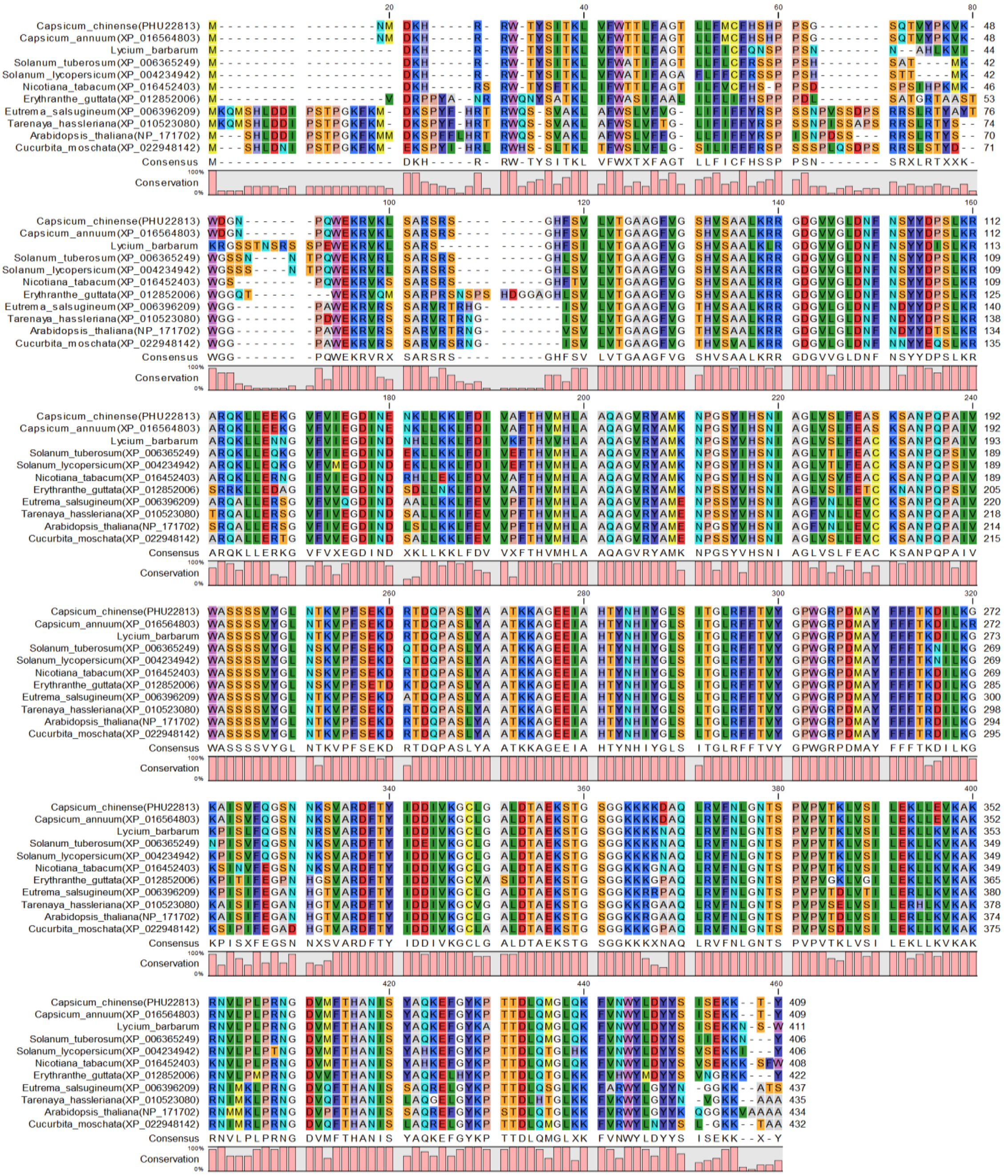
Capsicum_chinense(PHU22813)
Capsicum_annuum(XP_016564803)
Lycium_barbarum
Solanum_tuberosum(XP_006365249)
Solanum_lycopersicum(XP_004234942)
Nicotiana_tabacum(XP_016452403)
Erythranthe_guttata(XP_012852006)
Eutrema_salsugineum(XP_006396209)
Tarenaya_hassleriana(XP_010523080)
Arabidopsis_thaliana(NP_171702)
Cucurbita_moschata(XP_022948142)
Consensus
Conservation

(b)

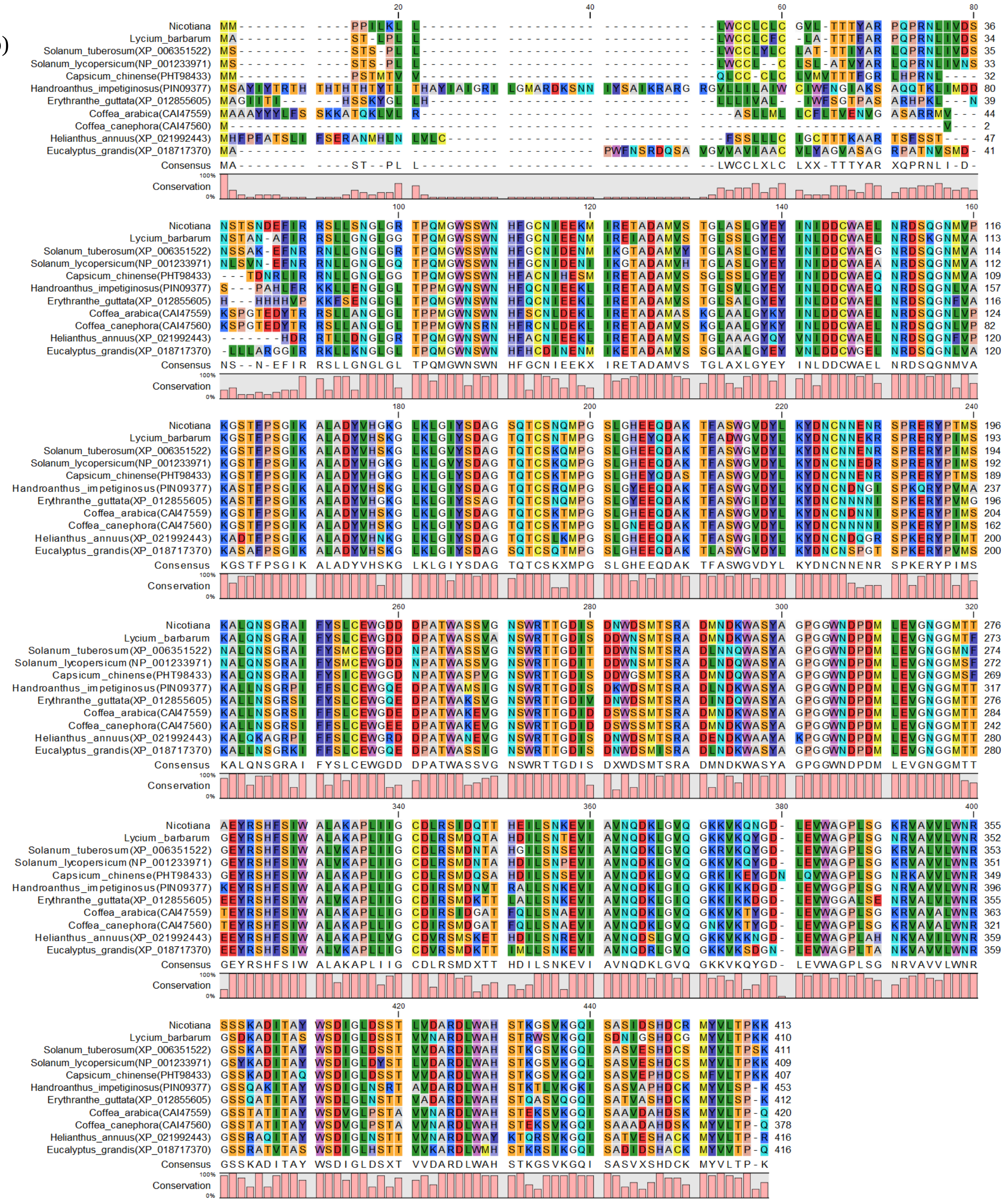
Nicotiana
Lycium_barbarum
Solanum_tuberosum(XP_006351522)
Solanum_lycopersicum(NP_001233971)
Capsicum_chinense(PHT98433)
Handroanthus_impetiginosus(PIN09377)
Erythranthe_guttata(XP_012855605)
Coffea_arabica(CAI47559)
Coffea_canephora(CAI47560)
Helianthus_annuus(XP_021992443)
Eucalyptus_grandis(XP_018717370)
Consensus
Conservation

(c)

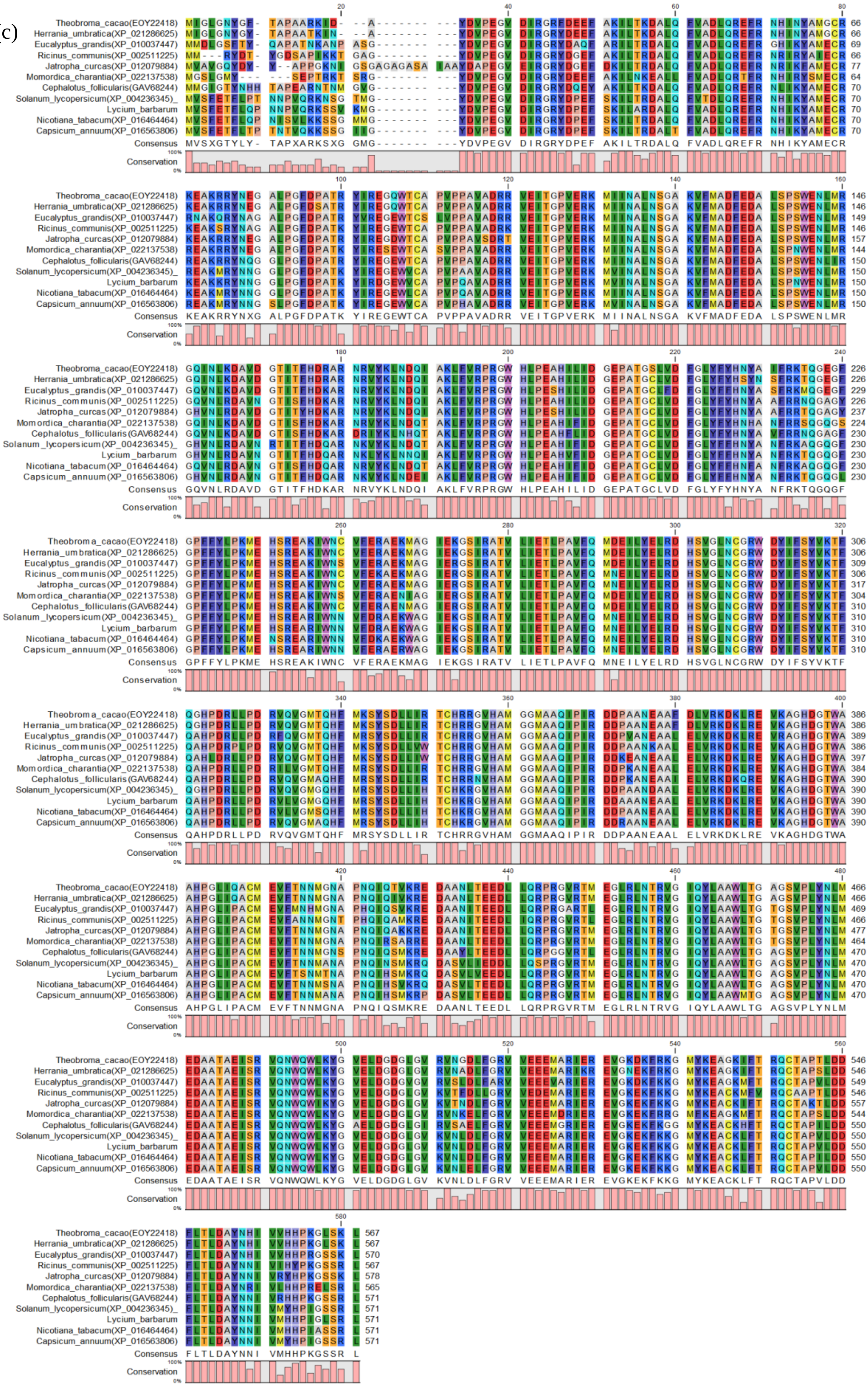
Theobroma_cacao(EOY22418)
Herrania_umbratica(XP_021286625)
Eucalyptus_grandis(XP_010037447)
Ricinus_communis(XP_002511225)
Jatropha_curcas(XP_012079884)
Momordica_charantia(XP_022137538)
Cephalotus_follicularis(GAV68244)
Solanum_lycopersicum(XP_004236345)_
Lycium_barbarum
Nicotiana_tabacum(XP_016464464)
Capsicum_annuum(XP_016563806)
Consensus
Conservation

Figure S3 Multiple sequence alignment of *LBGAE* (**a**), *LBGALA* (**b**) and *LBMS* (**c**)amino acid sequences with other known species.

Table S1 Primer sequences for ORF sequences cloning and qPCR

Gene name	Primer name	Primer sequence（5′-3′）RACE	TM（°C）
LBGAE	GSP1	GACTTGCTGGTTGATCCGTTCGAT	58
	GSP2	CCACGAGGAATGCATCAAACGCTTG	58
LBGALA	GSP1	GATTCGTCCACTGTTGTTAATGCACG	61
	GSP2	CCCAAAAGGCTTCTGCGGATGAACG	64
LBGAE	F2	ATGGACAAGCACCGTAGATGGAC	60
	R2	TCACCAAGAATTCTTCTTTTCACTTATTG	55
LBGALA	F2	GCCACGTCTAGTCTGCCCTTTCACT	60
	R2	CCTTCAGCAATGTAGTATTTTTCAC	55
Actin	F	CCATCTACGAGGGTTACGCTTTG	62
	R	AGTCAAGAGCCACATAGGCAAGC	62
LBGAE	F3	CGGCCTGATATGGCGTATTT	62
	R3	TCCCTTGCTACACTTCTATTATTGG	62
LBGALA	F4	CAATATGGAGACTTGGAGGTATGG	62
	R4	CAGCCTTGTCTGATCCTCTATTC	62
LBMS	F5	GCTGGTTCTGTCCCACTTTA	62
	R5	TCCATCCAATTCCACTCCATATT	62
